# *Ocimum gratissimum* leaf extract may precipitate infertility in male diabetic Wistar rats

**DOI:** 10.5935/1518-0557.20180072

**Published:** 2019

**Authors:** Shehu-Tijani Shittu, Seyyid Alli Shittu, Afeez Ayobami Olatunji, Wahab Adekunle Oyeyemi

**Affiliations:** 1 Department of Physiology, College of Medicine, University of Ibadan, Ibadan, Nigeria; 2 Department of Physiology, Igbinedion University, Okada, Edo State, Nigeria

**Keywords:** *Ocimum gratissimum*, fructosamine, sperm quality, testicular cytoarchitecture

## Abstract

**Objective:**

This study was designed to investigate the *Ocimum gratissimum* (OG) effects on sperm quality and testicular cytoarchitecture in alloxan-induced diabetic rats.

**Method:**

Twenty male Wistar rats (150-200 g) were assigned into 4 groups (n=5) as A (control), B (OG), C (Dia) and D (Dia+OG). Groups A and B were normal animals receiving distilled water or OG (400 mg/kg), respectively while diabetes was induced by alloxan monohydrate (100 mg/kg) in groups C and D, followed by the administration of distilled water or OG, respectively for 28 days. Blood samples were obtained for fasting blood glucose (FBG) and fructosamine determination while, epididymis and testes were obtained for sperm quality assessment using computer-assisted sperm analyzer and testicular histomorphometry, respectively. Seminiferous tubule diameter and interstitial space distance were quantified in hematoxylin and eosin stained slides. Statistical analysis was done using ANOVA and student t-test at α0.05.

**Results:**

Fructosamine and FBG were reduced in Dia+OG (80.11±3.80µmol/L and 132.0±8.41mg/dl, respectively) compared with Dia (139.66±4.29µmol/L and 285.6±26.69mg/dl, respectively). Sperm count was unchanged in Dia, but decreased in OG and Dia+OG; abnormal sperm cells increased in OG, Dia and Dia+OG. Mild vacuolation in the seminiferous tubule, disorganized germinal cells layer, arrested sperm maturation with empty spermatozoa in lumen, decreased seminiferous tubule diameter and increased interstitial space were found in the testes of OG, Dia and Dia+OG compared with control.

**Conclusion:**

Diabetes induces sperm impairments and distortions in testicular cytoarchitecture, which were aggravated by OG leaf extract in male Wistar rats.

## INTRODUCTION

Sexual dysfunctions ranging from erectile and testicular dysfunctions, reduced libido, retrograde ejaculation (Dunsmuir & Holmes, 1996), disrupted endocrine control of spermatogenesis (Baccetti *et al*., 2002), impaired sperm DNA integrity (Agbaje *et al*., 2007), reduced sperm count and motility (Barták, 1979; Bhattacharya *et al*., 2014) and low serum testosterone (Verma *et al*., 2013) are complications of prolonged diabetes mellitus in men. Thus, a high prevalence of infertility and subfertility is associated with type 1 and type 2 diabetes mellitus (Alves & Oliveira, 2013; Rato *et al*., 2013). The prevalence could be as high as 35% in type 2 diabetes mellitus (La Vignera *et al*., 2012a), with about 90% of diabetics experiencing sexual upheavals such as decreased libido, impotence and infertility (Corona *et al*., 2014). With the growing incidence of diabetes mellitus, which had already surpassed the World Health Organization’s projection for 2025 by 28.9% in 2014 (Temidayo & Stefan, 2018), infertility among diabetic men is likely to grow in similar progression.

Data from experimental diabetes mellitus have also shown that uncontrolled hyperglycaemia may be deleterious to the male reproductive function. For instance, decreased copulatory behavior (Scarano *et al*., 2006), reduced fertility, decrease Leydig cells population (Ballester *et al*., 2004) and increased testicular toxicity (Hadi *et al*., 2013; Iweala *et al*., 2013) were reported in diabetic rats. Also, in prediabetes induced by high energy diet in rats, alterations in testicular glucose metabolism and epididymal bicarbonate dynamics may adversely affect sperm storage and viability (Rato *et al*., 2013). The testicular dysfunction/degeneration in experimental diabetes was hypothesized to involve oxidative stress (Amaral *et al*., 2006; Shrilatha & Muralidhara, 2007; La Vignera *et al*., 2012b). Increased oxidative stress is associated with increased sperm DNA damage and spermatogenic gene expression (Mallidis *et al*., 2007; 2009); hence, treatment with different antioxidants were shown to improve diabetes-induced sperm abnormality in animal models (Rabbani *et al*., 2009; Bal *et al*., 2011; Mohasseb *et al*., 2011; Shi *et al*., 2017; Tsounapi *et al*., 2018) and men (Omu *et al*., 2014). The lack of insulin stimulatory effect on Follicle Stimulating Hormone (FSH) in experimental type-1 diabetes may also play a role in the decreased Leydig cell function and testosterone production (Ballester *et al*., 2004); given that insulin replacement significantly improved sperm quality and testicular cytoarchitecture in diabetic rats (Seethalakshmi *et al*., 1987; Soudamani *et al*., 2005).

*Ocimum gratissimum* (OG), planted in Nigeria for its nutritional and medicinal value, has been shown by several researchers to possess hypoglycaemic (Aguiyi *et al*., 2000; Owoyele *et al*., 2005; Egesie *et al*., 2006) and antioxidant (Akinmoladun *et al*., 2007; Aprioku & Obianime, 2008; Shittu *et al*., 2016) properties. The hypoglycemic property was associated with inhibition of hepatic glycogen phosphorylase activity in streptozotocin-induced diabetic rats (Shittu et al., 2018). Current evidences in normal rats (Leigh & Fayemi, 2008) and mice (Obianime *et al*., 2010) have documented that OG may possess anti-fertility properties in a dose and duration dependent manner. However, there are conflicting reports on the influence of OG on male reproductive parameters in diabetic rats. For instance, Arfa & Rashed (2008) reported an elevated testosterone level, while Ebong *et al*. (2014) observed no changes in reproductive hormones in OG-treated diabetic rats. Also, Asuquo *et al.* (2009) reported improvements in testicular morphology, while Onuka *et al.* (2014) reported impaired sperm parameters in OG-treated diabetic rats. It is therefore pertinent to investigate the effects of OG on sperm quality and testicular cytoarchitecture in alloxan induced diabetic rats.

## MATERIALS AND METHODS

### Animals

Twenty male Wistar rats were obtained from the Central animal house, College of Medicine - University of Ibadan, Ibadan. They were housed and acclimatized for two weeks in the Department of Physiology animal house, University of Ibadan, under standard laboratory conditions with natural photoperiod of 12 hours light: dark cycle. They were allowed free access to rat chow (Ladokun Feeds) and water. All experimental and handling protocols were in compliance with institutional ethical regulation and the NIH publication No. 85-23 guidelines (NIH publication revised, 1985).

### Preparation of aqueous leaf extract of *Ocimum gratissimum*

Fresh leaves of OG were obtained from the Bode market in the Ibadan metropolis. Identified and authenticated at the Forest Research Institute of Nigeria (FHI.110026). The fresh leaves were washed, air-dried and pulverized into powdery form. One kilogram of the powder was soaked in a glass container with distilled water for aqueous extraction for 24 hours, filtered and the filtrate was collected in a round bottom flask. The filtrate was evaporated using a rotary evaporator to yield 8.51% extract. The extract was administered at 400 mg/kg body weight which had been previously reported to be non-lethal (Aguiyi *et al*., 2000).

### Induction of diabetes mellitus

Diabetes mellitus was induced by intraperitoneal administration of 100 mg/kg body weight of alloxan monohydrate (Sigma^®^, St Louis, USA). Diabetes was confirmed after 72 hours using a One Touch Ultra glucometer^®^. Animals with fasting blood glucose ≥200mg/dl were considered diabetic.

### Experimental design

The rats were randomly divided into 4 groups (n=5) and treated per os for 28 days as follows:

- Control: Normal animals administered distilled water daily
- *Ocimum gratissimum* (OG): Normal animals administered 400 mg/kg of OG
- Diabetic Untreated (Dia): Alloxan-induced diabetic rats administered distilled water
- Diabetic Treated (Dia+OG): Alloxan-induced diabetic rats administered 400 mg/kg of OG

Fasting blood glucose (FBG) was monitored at the start and at the end of the 28 day- treatment. Blood sample for FBG was collected via the tail vein and measured using One Touch Ultra^®^ glucometer.

### Sample collection

After the 28-day treatment, under anesthesia induced by intraperitoneal administration of 50mg/kg sodium thiopental (Rotec Medica, Trittau, Germany), blood samples were collected via cardiac puncture for measuring fructosamine levels while the epididymis and testes were collected for sperm quality assessment and determination of testicular cytoarchitecture.

### Determination of serum fructosamine

Fructosamine levels were measured using the commercially available fructosamine kit (Fortress Diagnostics Limited^®^, United Kingdom). The colorimetric test principle is based on the ability of ketoamines to reduce nitrotetrazolium-blue to formazan in an alkaline solution. The rate of formazan formation is directly proportional to the fructosamine concentration. Uric acid interference is eliminated by Uricase and a detergent eliminates matrix effects. The rate of reaction is photometrically measured at 546nm. Briefly, 50µL of sample or calibrator was added to 1000µL working reagent made up of Potassium phosphate buffer, Nitrotetrazolium-blue, Sodium cholate, Potassium carbonate buffer (pH 10.3), Uricase (Arthrobacter species) and detergent. It was mixed and incubated for 9 minutes at 37ºC. The absorbance at 546 nm was recorded immediately (A1) and after exactly 60 seconds (A2). The fructosamine concentration (µmol/L) was calculated as:

$$=\frac{A2-A1\;\mathrm{Sample}}{A2-A1\;\mathrm{Calibrator}}\times \mathrm{Concentration}\;\mathrm{of}\;\mathrm{Calibrator}$$

### Sperm Count

The right epididymis was placed in a pre-warmed (37ºC) Petri dish containing 2mL of phosphate buffer saline solution (pH 7.4). The caudal portion was punctured twice with the tip of a scalpel to release sperm, commencing a 3-minute “swim-out” period. After the swim-out, the dish was gently swirled, and a 9µL sample from a relatively dense portion of the sperm cloud was placed onto a counting chamber. The sperm count (millions/ml) was determined using a computer-aided sperm analyser (CASA, JH-6004 Sperm Quality Analyser).

### Sperm Morphology

The left caudal epididymis was weighed, minced in a Petri dish containing 2 mL of deionized water and swirled gently. A drop of the dish content was placed on a standard glass microscope slide. The edge of a clean slide was gently dragged across the drop to make a thin layer of sperm cells which was put to air dry. The air-dried sperm smeared slide was fixed in 95% and 50% (v/v) ethanol for 15 minutes and 30 seconds, respectively; then rinsed in distilled water for 30 seconds and stained with Harris’s hematoxylin and G-6 orange for 4 minutes and 1 minute, respectively. It was then dipped in ethanol 95% for 2 minutes, before staining with EA-50 green for 1 minute (Papanicolaou). The stained slide was then immersed in a xylene and ethanol mixture in a ratio of 1 to 2, and in 100% xylene for 1 minute each, respectively. It was then drained for 1-2 seconds (Marshall, 1983). Two slides were made from each caudal epididymal tissue. The stained slides were examined under the microscope with a x100-objective lens and ×10 eyepieces. Abnormal sperm cells were counted and expressed as a percentage.

### Testicular histomorphometry

The testes were immediately fixed in Bouin’s fluid for 24 hours. It was subsequently dehydrated twice in ascending grade of absolute alcohol for one hour, and placed in xylene for 1 hour for clearing. After removing it from xylene, it was placed in a wax bath for one hour and embedded in paraffin wax. It was then trimmed and sectioned at 3-5 micron slices with a microtome. The section was floated with 20% alcohol on water at a temperature of 5ºC (below paraffin wax melting point), picked with a clean grease-free microscope slide and drained for 1 hour. It was then stained with hematoxylin and eosin and mounted. Photomicrographs of the sections were made to observe general morphology and morphological changes. Seminiferous diameter and interstitial space distance were quantified by imageJ software (version 1.49, National Institutes of Health, Bethesda, MD, USA, http://rsb.info.nih.gov/ij/).

### Statistical analysis

The data from each group was expressed as mean ± standard error of the mean (mean±SEM). The data was analyzed using ANOVA and Student t-test. *p*<0.05 was considered significant. All analyzes were performed using GraphPad prism, version 7.

## RESULTS

### Effect of *Ocimum gratissimum* on fasting blood glucose and serum fructosamine

**Figure 1:**
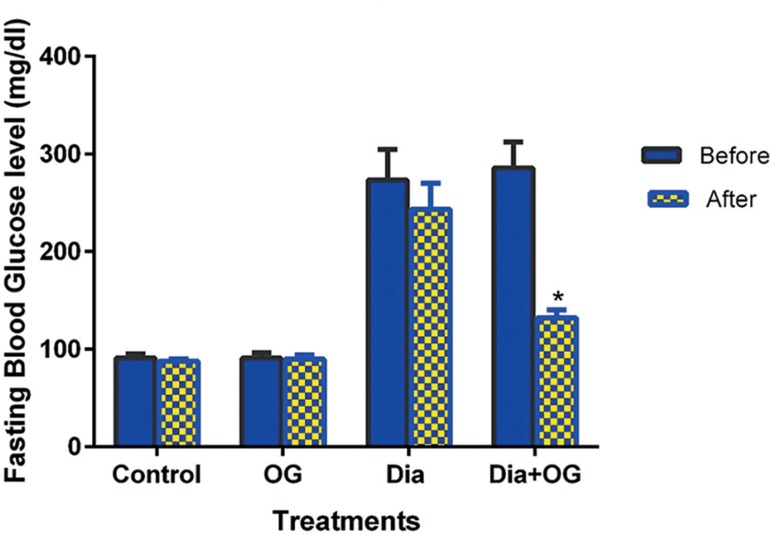
Effects of *Ocimum Gratissimum* on fasting blood glucose level in normal and diabetic male rats. n=5, *p<0.05 Before vs. After.

**Figure 2:**
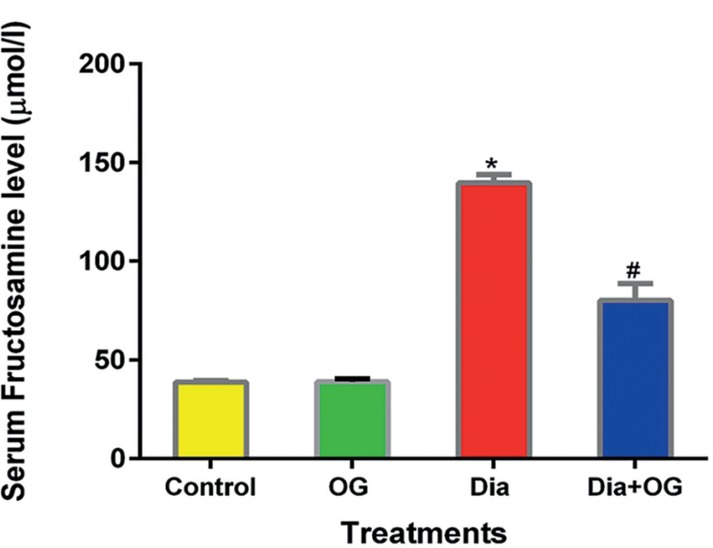
Effects of *Ocimum Gratissimum* on Serum fructosamine in normal and diabetic male rats. n=5, *p<0.05 Control vs. Dia; #*p*<0.05 Dia *vs.* Dia+OG.

As shown in Figure 1, there was no significant difference in the blood glucose levels of the control and the OG-treated normal animals [Control (90.88±4.29 mg/dl vs. 87.75±2.20mg/dl); OG (90.83±5.49 mg/dl vs. 89.67±4.21 mg/dl)] before and after the 28-day treatment. However, fasting blood glucose level was significantly reduced (α0.05) from 285.6±26.69mg/dl before treatment to 132.0±8.41mg/dl after treatment (*p*<0.05) in the Dia+OG. The effect of OG on fructosamine is shown in Figure 2. Fructosamine level was significantly increased (α0.05) in the Dia group (139.66±4.29µmol/L) when compared with the controls (38.71±0.85µmol/L); while it was decreased (α0.05) in the Dia+OG (80.11±3.80µmol/L) compared with Dia (139.66±4.29µmol/L).

### Effect of *Ocimum gratissimum* sperm quality

**Figure 3:**
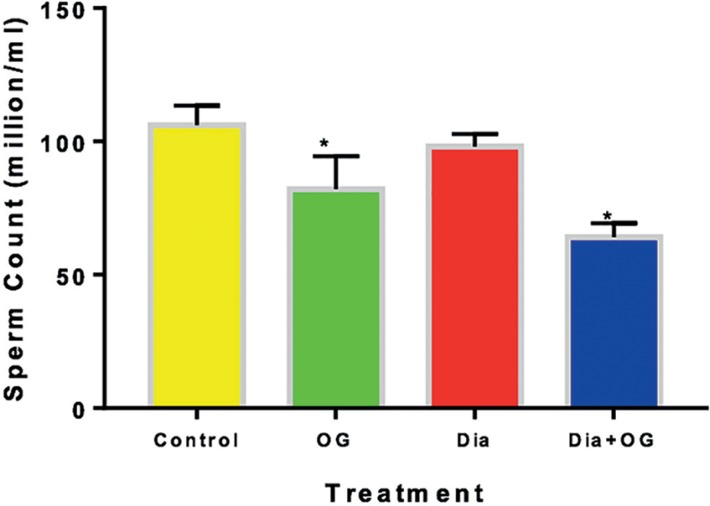
Effects of *Ocimum gratissimum* on sperm counts in normal and diabetic male rats. n=5, *p<0.05 compared with Control animals.

**Figure 4:**
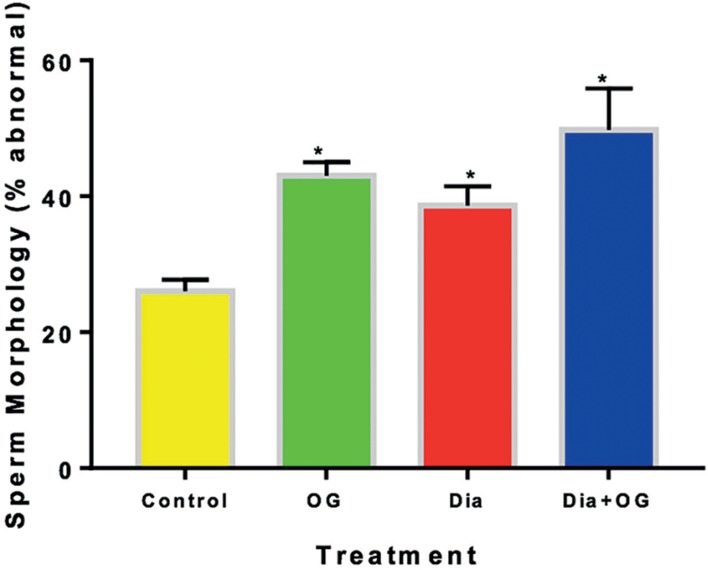
Effect of *Ocimum gratissimum* on sperm morphology in normal and diabetic male rats. n=5, *p<0.05 compared with Control animals.

Sperm count decreased significantly (α0.05) in OG, Dia and Dia+OG when compared with the controls (Figure 3). The sperm counts in the Dia group was significantly higher (α0.05) than the counts in the Dia+OG group. As shown in Figure 4, the percentage of abnormal sperm morphology was significantly increased (α0.05) in normal rats treated with OG (42.67±1.67%), diabetic untreated rats (38±1.63%) and diabetic rats treated with OG (46.50±5.95%), when compared with the controls (25.83±1.11%).

### Effects of *Ocimum gratissimum* on testicular histomorphometry

**Figure 5:**
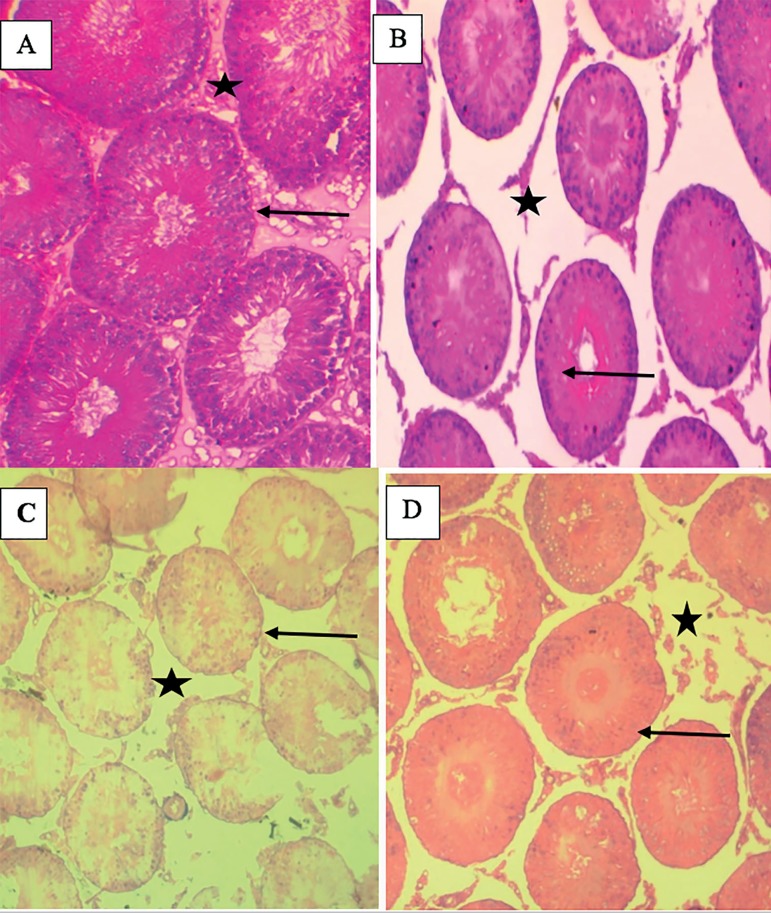
Photomicrographs of the testes showing seminiferous tubules (black arrow) and their respective interstitial space (black star box) in (A), Control group shows normal seminiferous tubule with complete sperm maturation, normal germinal cells layer, presence of spermatozoa strand in the lumen and interstitial cells appear normal. (B) OG (C) Dia and (D) Dia+OG groups show mild vacuolation in the seminiferous tubule, disorganized germinal cells layer, arrest sperm maturation with empty spermatozoa in lumen. X 100.

The effects of OG on testicular cytoarchitecture of normal and diabetic rats are shown in Figure 5 and Table 1. Mild vacuolation in the seminiferous tubule, disorganized germinal cells layer, arrested sperm maturation with empty spermatozoa in lumen where observed in OG, Dia and Dia+OG animals when compared with their control counterparts. The seminiferous tubule diameter was significantly decreased (α0.05) in OG, Dia and Dia+OG groups when compared with the control animals; while interstitial space distance was significantly increased (α0.05) in OG, Dia, and Dia+OG when compared with the control animals.

**Table 1:** Effects of *Ocimum gratissimum* on seminiferous tubular diameter and testicular interstitial space distance in normal and diabetic rats.

	Seminiferous tubular diameter (µm)	Testicular interstitial space distance (µm)
Control	492.79±24.50	34.09±3.80
OG	137.39±4.62 *	66.69±15.86 *
Dia	153.41±7.43 *	58.66±5.23 *
Dia+OG	172.88±8.53 *	56.56±13.97 *

* *p*<0.05 when compared with the control.

## DISCUSSION

The objective of the present study was to evaluate the effects of OG on sperm quality and testicular cytoarchitecture in male diabetic rats. The reduction in the fasting blood glucose level observed in the diabetic animals treated with OG is consistent with the reported hypoglycemic effect of OG (Aguiyi *et al.*, 2000; Owoyele *et al.*, 2005; Egesie *et al.*, 2006; Mohammed *et al.*, 2007; Oguanobi *et al.*, 2012; Okoduwa *et al*., 2017). Fructosamine was quantified in the present study to monitor glycemic control in the diabetic rats. Fructosamine is formed by the glycation of primary amine and its subsequent isomerization via the Amadori rearrangement (Armbruster, 1987). It reflects glycemic control over the previous 2-4 weeks (Nagasaka *et al*., 1988), without any influence of erythrocyte diseases (Koga *et al*., 2011). Although fructosamine is yet to gain wider use, like glycated hemoglobin (HbA1c) in monitoring diabetes control (Nansseu *et al*., 2015), studies are pointing to its tendency of outperforming HbA1c (Rendell *et al*., 1986; Misciagna *et al*., 2004). The elevated fructosamine level in the diabetic rats of the present study is in line with the documented effect of experimental diabetes on fructosamine (Petlevski *et al*., 2001; You *et al*., 2015; Ren *et al*., 2017). To the best of our knowledge, this present study is the first to report the effects of OG on fructosamine levels in diabetic rats. Decrease fructosamine is linearly associated with glycemic control (van Eijk *et al*., 2007); accordingly, fructosamine levels decreased with decrease in fasting blood glucose level in the OG-treated animals in the present study.

The decreased sperm count and increased percentage of abnormal sperm cells in all the OG-treated animals of this study indicate that OG poses anti-fertility effects on normal and diabetic rats. This is consistent with the report of Leigh & Fayemi (2008) that aqueous extract of OG has deleterious effects on both spermatogenesis and maturation of spermatozoa at different stages of germ cell development. Nevertheless, impairment in these sperm parameters were also observed in the diabetic untreated group, it is more pronounced in the OG-treated diabetic animals. It is a well-documented phenomenon in which increased sperm abnormality is an indicator of testicular pathology (Câmara *et al*., 2014).

The decreased mean diameter of seminiferous tubules in the experimental animals suggests that testicular damage caused by OG could be linked to the observed significant changes in sperm parameters, since the histological integrity of the entire testis is fundamental to the production of fertile spermatozoa (White, 1933). Findings from the present study is in line with the previous reports that OG caused distortion and destruction of the architecture and structure of the testicular histology with varying degrees of edema within the interstitial cells in normal mice (Obianime *et al.*, 2010). Reduction in tubular size is associated with detachment and loss of germ cells, which is observed in the testis of rats treated with different drugs (Sasso-Cerri & Miraglia, 2002). Reduction in tubular size is also characterized with structural injury to the Sertoli cells, which disrupts the Sertoli cell-germ cell physical interaction (Richburg & Boekelheide, 1996) and induces programmed cell death among the detached germ cells (Richburg *et al*., 1999).

Gonadal stem cell damage with low sperm counts or azoospermia, which depends on treatment regimen, route and dose, have been observed with anticancer agents (Lee *et al*., 2006). A concentration-dependent anti-proliferative activity of OG was documented in prostate cancer (PC-3) cells *in-vitro* (Ekunwe *et al*., 2010). Aqueous leaf extract of OG also inhibited proliferation, migration, anchorage independent growth, morphogenesis, induction of COX-2 protein (Nangia-Makker *et al*., 2007) and matrix metaloproteases (Nangia-Makker *et al*., 2013) in breast cancer cells. Such tumor prevention potential is common in plants with polyphenolic compounds, anti-oxidants, vitamins, and v-3 fatty acids (Fahey *et al*., 1997; Pezzuto, 1997; Kobayashi *et al*., 2000); it is therefore not surprising that polyphenolic and antioxidant components were reported in OG (Venuprasad *et al*., 2014). Hence, the anticancer activity of OG might have roles to play in its degenerative and deleterious effects on testicular cytoarchitecture and adverse changes in sperm parameters.

Findings from this study showed that although the hypoglycaemic effect of OG is evident with the decreased fructosamine level in diabetic rats; it has debilitating effects on male fertility characterized by reduction in sperm count, increased percentage of abnormal sperm morphology and distortions in testicular cytoarchitecture, which are worsened by diabetes mellitus. Thus, it is important to isolate the active hypoglycaemic component of *Ocimum gratissimum* to harness it beneficial usage in diabetes mellitus.

## References

[r1] Agbaje IM, Rogers DA, McVicar CM, McClure N, Atkinson AB, Mallidis C, Lewis SE (2007). Insulin dependant diabetes mellitus: implications for male
reproductive function. Hum Reprod.

[r2] Aguiyi JC, Obi CI, Gyang SS, Igweh AC (2000). Hypoglycaemic activity of Ocimum gratissimum in
rats. Fitoterapia.

[r3] Akinmoladun AC, Ibukun EO, Afor E, Obutor EM, Farombi EO (2007). Phytochemical constituents and antioxidant activity of extract
from the leaves of ocimum gratissimum. Sci Res Essay.

[r4] Alves MG, Oliveira PF (2013). Diabetes mellitus and male reproductive function: where we
stand?. Int J Diabetol Vasc Dis Res.

[r5] Amaral S, Moreno AJ, Santos MS, Seiça R, Ramalho-Santos J (2006). Effects of hyperglycemia on sperm and testicular cells of
Goto-Kakizaki and streptozotocin-treated rat models for
diabetes. Theriogenology.

[r6] Aprioku JS, Obianime AW (2008). Antioxidant Activity of the Aqueous Crude Extract of Ocimum
gratissimum LINN. Leaf on Basal and Cadmium-induced Plasma Levels of
Phosphatases in Male Guinea-pigs. J Appl Sci Environ Manage.

[r7] Arfa MM, Rashed AM (2008). The modulative biochemical effect of extract of ocimum
gratissimum as anti-oxidant on diabetic albino rats. Egypt J Comp Path Clinic Path.

[r8] Armbruster DA (1987). Fructosamine: structure, analysis, and clinical
usefulness. Clin Chem.

[r9] Asuquo O, Edet A, Mesembe O, Atanghwo J (2009). Ethanolic Extracts of Vernonia Amygdalina and Ocimum Gratissimum
Enhance Testicular Improvement in Diabetic Wistar Rats. Internet J Altern Med.

[r10] Baccetti B, La Marca A, Piomboni P, Capitani S, Bruni E, Petraglia F, De Leo V (2002). Insulin-dependent diabetes in men is associated with
hypothalamo-pituitary derangement and with impairment in semen
quality. Hum Reprod.

[r11] Bal R, Türk G, Tuzcu M, Yilmaz O, Ozercan I, Kuloglu T, Gür S, Nedzvetsky VS, Tykhomyrov AA, Andrievsky GV, Baydas G, Naziroglu M (2011). Protective effects of nanostructures of hydrated C(60) fullerene
on reproductive function in streptozotocin-diabetic male
rats. Toxicology.

[r12] Ballester J, Muñoz MC, Domínguez J, Rigau T, Guinovart JJ, Rodríguez-Gil JE (2004). Insulin-dependent diabetes affects testicular function by FSH-
and LH-linked mechanisms. J Androl.

[r13] Barták V (1979). Sperm quality in adult diabetic men. Int J Fertil.

[r14] Bhattacharya SM, Ghosh M, Nandi N (2014). Diabetes mellitus and abnormalities in semen
analysis. J Obstet Gynaecol Res.

[r15] Câmara LBRM, Câmara DR, Maiorino FC, Silva Júnior VA, Guerra MMP (2014). Canine testicular disorders and their influence on sperm
morphology. Anim Reprod.

[r16] Corona G, Giorda CB, Cucinotta D, Guida P, Nada E, Gruppo di studio SUBITO-DE (2014). Sexual dysfunction at the onset of type 2 diabetes: the interplay
of depression, hormonal and cardiovascular factors. J Sex Med.

[r17] Dunsmuir WD, Holmes SA (1996). The aetiology and management of erectile, ejaculatory, and
fertility problems in men with diabetes mellitus. Diabet Med.

[r18] Ebong PE, Efiong EE, Mgbeje BIA, Igile GO, Itam EH (2014). Combined Therapy of Moringa oleifera and Ocimum gratisimum
Reversed Testicular Damage in Diabetic Rats. Brit J Med Med Res.

[r19] Egesie UG, Adelaiye AB, Ibu JO, Egesie OJ (2006). Safety and hypoglycaemic properties of aqueous leaf extract of
Ocimum gratissimum in streptozotocin induced diabetic rats. Niger J Physiol Sci.

[r20] Ekunwe SI, Thomas MS, Luo X, Wang H, Chen Y, Zhang X, Begonia GB (2010). Potential cancer-fighting Ocimum gratissimum (OG) leaf extracts:
increased anti-proliferation activity of partially purified fractions and
their spectral fingerprints. Ethn Dis.

[r21] Fahey JW, Zhang Y, Talalay P (1997). Broccoli sprouts: an exceptionally rich source of inducers of
enzymes that protect against chemical carcinogens. Proc Natl Acad Sci U S A.

[r22] Hadi MA, Zaidan HK, Natah TM, Al-Saadi AH (2013). Protective Effect of Plants Extracts Mixture on Sperm
Abnormalities, Testicular and Epididymal Tissues in Diabetic Male
Rats. J Nat Sci Res.

[r23] Iweala EEJ, Uhegbu FO, Adesanoye OA (2013). Biochemical effects of leaf extracts of Gongronema latifolium and
selenium supplementation in alloxan induced diabetic rats. J Pharmacognosy Phytother.

[r24] Kobayashi T, Nakata T, Kuzumaki T (2000). Effect of flavonoids on cell cycle progression in prostate cancer
cells. Cancer Lett.

[r25] Koga M, Hashimoto K, Murai J, Saito H, Mukai M, Ikegame K, Ogawa H, Kasayama S (2011). Usefulness of glycated albumin as an indicator of glycemic
control status in patients with hemolytic anemia. Clin Chim Acta.

[r26] La Vignera S, Condorelli R, Vicari E, D’Agata R, Calogero AE (2012a). Diabetes mellitus and sperm parameters. J Androl.

[r27] La Vignera S, Condorelli RA, Vicari E, D'Agata R, Salemi M, Calogero AE (2012b). High levels of lipid peroxidation in semen of diabetic
patients. Andrologia.

[r28] Lee SJ, Schover LR, Partridge AH, Patrizio P, Wallace WH, Hagerty K, Beck LN, Brennan LV, Oktay K, American Society of Clinical Oncology (2006). American Society of Clinical Oncology recommendations on
fertility preservation in cancer patients. J Clin Oncol.

[r29] Leigh OL, Fayemi OE (2008). Effects of crude aqueous extract of Ocimum gratissium leaves on
testicular histology and spermiogram in male albino rats (Wistar
strain). Vet Res.

[r30] Mallidis C, Agbaje I, Rogers D, Glenn J, McCullough S, Atkinson AB, Steger K, Stitt A, McClure N (2007). Distribution of the receptor for advanced glycation end products
in the human male reproductive tract: prevalence in men with diabetes
mellitus. Hum Reprod.

[r31] Mallidis C, Agbaje I, O'Neill J, McClure N (2009). The influence of type 1 diabetes mellitus on spermatogenic gene
expression. Fertil Steril.

[r32] Marshall PN (1983). Papanicolaou staining--a review. Microsc Acta.

[r33] Misciagna G, Logroscino G, De Michele G, Cisternino AM, Guerra V, Freudenheim JL (2004). Fructosamine, glycated hemoglobin, and dietary
carbohydrates. Clin Chim Acta.

[r34] Mohammed A, Tanko Y, Okasha MA, Magaji RA, Yaro AH (2007). Effects of aqueous leaves extract of Ocimum gratissimum on blood
glucose levels of streptozotocin-induced diabetic wistar
rats. Afr J Biotechnol.

[r35] Mohasseb M, Ebied S, Yehia MA, Hussein N (2011). Testicular oxidative damage and role of combined antioxidant
supplementation in experimental diabetic rats. J Physiol Biochem.

[r36] Nagasaka Y, Fujii S, Yaga K, Matsumura S, Kaneko T (1988). Clinical Application of Measuring Serum Fructosamine as an Index
of Glycemic Control in Diabetic Patients. Bull Yamaguchi Med Sch.

[r37] Nangia-Makker P, Tait L, Shekhar MP, Palomino E, Hogan V, Piechocki MP, Funasaka T, Raz A (2007). Inhibition of breast tumor growth and angiogenesis by a medicinal
herb: Ocimum gratissimum. Int J Cancer.

[r38] Nangia-Makker P, Raz T, Tait L, Shekhar MP, Li H, Balan V, Makker H, Fridman R, Maddipati K, Raz A (2013). Ocimum gratissimum retards breast cancer growth and progression
and is a natural inhibitor of matrix metalloproteases. Cancer Biol Ther.

[r39] Nansseu JR, Fokom-Domgue J, Noubiap JJ, Balti EV, Sobngwi E, Kengne AP (2015). Fructosamine measurement for diabetes mellitus diagnosis and
monitoring: a systematic review and meta-analysis protocol. BMJ Open.

[r69] NIH - National Institue of Health. U.S., Department of Health an human Services NIH Publication No 86-23 (revised 1985): Guide for the Care and Use of
Laboratory Animals.

[r40] Obianime AW, Aprioku JS, Esomonu CTO (2010). Antifertility effects of aqueous crude extract of Ocimum
gratissimum L. leaves in male mice. J Med Plant Res.

[r41] Oguanobi NI, Chijioke CP, Ghasi S (2012). Anti-diabetic effect of crude leaf extracts of Ocimum gratissimum
inneonatal streptozotocin-induced type-2 model diabetic rats. Int J Pharm Pharm Sci.

[r42] Okoduwa SIR, Umar IA, James DB, Inuwa HM (2017). Anti-Diabetic Potential of Ocimum gratissimum Leaf Fractions in
Fortified Diet-Fed Streptozotocin Treated Rat Model of Type-2
Diabetes. Medicines (Basel).

[r43] Omu AE, Al-Bader MD, Al-Jassar WF, Al-Azemi MK, Omu FE, Mathew TC, Anim JT (2014). Antioxidants Attenuates the Effects of Insulin Dependent Diabetes
Mellitus on Sperm Quality. Bioenergetics.

[r44] Onuka AE, Mounmbegna PE, Nwafor A (2014). Polyherbal Extract of Ocimum Gratissimum and Gongronema
Latifolium on Reproductive Functions in Alloxan Induced Diabetic Male
Rats. J Med Sci Clin Res.

[r45] Owoyele BV, Funsho MA, Soladoye AO (2005). Effect of aqueous leaves extract of Ocimum gratissimum (sweet
basil) on alloxan induced diabetic rats. Phcog Mag.

[r46] Petlevski R, Hadzija M, Slijepcevic M, Juretic D (2001). Effect of ‘antidiabetis’ herbal preparation on serum glucose and
fructosamine in NOD mice. J Ethnopharmacol.

[r47] Pezzuto JM (1997). Plant-derived anticancer agents. Biochem Pharmacol.

[r48] Rabbani SI, Devi K, Khanam S (2009). Inhibitory effect of glimepiride on nicotinamide-streptozotocin
induced nuclear damages and sperm abnormality in diabetic Wistar
rats. Indian J Exp Biol.

[r49] Rato L, Alves MG, Dias TR, Lopes G, Cavaco JE, Socorro S, Oliveira PF (2013). High-energy diets may induce a pre-diabetic state altering
testicular glycolytic metabolic profile and male reproductive
parameters. Andrology.

[r50] Ren T, Zhu Y, Xia X, Ding Y, Guo J, Kan J (2017). Zanthoxylum alkylamides ameliorate protein metabolism disorder in
STZ-induced diabetic rats. J Mol Endocrinol.

[r51] Rendell M, Paulsen R, Eastberg S, Stephen PM, Valentine JL, Smith CH, Nierenberg J, Rasbold K, Klenk D, Smith PK (1986). Clinical use and time relationship of changes in affinity
measurement of glycosylated albumin and glycosylated
hemoglobin. Am J Med Sci.

[r52] Richburg JH, Boekelheide K (1996). Mono-(2-ethylhexyl) phthalate rapidly alters both Sertoli cell
vimentin filaments and germ cells apoptosis in young rat
testes. Toxicol Appl Pharmacol.

[r53] Richburg JH, Nañez A, Gao H (1999). Participation of the Fas-signaling system in the initiation of
germ cell apoptosis in young rat testes after exposure to
mono-(2-ethylhexyl) phthalate. Toxicol Appl Pharmacol.

[r54] Sasso-Cerri E, Miraglia SM (2002). In situ demonstration of both TUNEL-labeled germ cell and Sertoli
cell in the cimetidine-treated rats. Histol Histolopathol.

[r55] Scarano WR, Messias AG, Oliva SU, Klinefelter GR, Kempinas WG (2006). Sexual behaviour, sperm quantity and quality after short-term
streptozotocin-induced hyperglycaemia in rats. Int J Androl.

[r56] Seethalakshmi L, Menon M, Diamond D (1987). The effect of streptozotocin-induced diabetes on the
neuroendocrine-male reproductive tract axis of the adult rat. J Urol.

[r57] Shi GJ, Zheng J, Wu J, Qiao HQ, Chang Q, Niu Y, Sun T, Li YX, Yu JQ (2017). Beneficial effects of Lycium barbarum polysaccharide on
spermatogenesis by improving antioxidant activity and inhibiting apoptosis
in streptozotocin-induced diabetic male mice. Food Funct.

[r58] Shittu ST, Oyeyemi WA, Lasisi TJ, Shittu SA, Lawal TT, Olujobi ST (2016). Aqueous leaf extract of Ocimum gratissimum improves hematological
parameters in alloxan-induced diabetic rats via its antioxidant
properties. Int J App Basic Med Res.

[r59] Shittu ST, Oyeyemi WA, Shittu SA, Lasisi TJ (2018). Ocimum gratissimum inhibits glycogen phosphorylase activity
without changes in hepatic nuclear factor kappa B (NF-kB) and inducible
nitric oxide synthase (iNOS) in streptozotocin-induced diabetic
rats. Niger Med Pract.

[r60] Shrilatha B, Muralidhara (2007). Early oxidative stress in testis and epididymal sperm in
streptozotocin-induced diabetic mice: its progression and genotoxic
consequences. Reprod Toxicol.

[r61] Soudamani S, Malini T, Balasubramanian K (2005). Effects of streptozotocin-diabetes and insulin replacement on the
epididymis of prepubertal rats: histological and histomorphometric
studies. Endocr Res.

[r62] Temidayo SO, Stefan SP (2018). Diabetes mellitus and male infertility. Asian Pac J Reprod.

[r63] Tsounapi P, Honda M, Dimitriadis F, Shimizu S, Shiomi T, Hikita K, Saito M, Tomita S, Sofikitis N, Takenaka A (2018). Antioxidant treatment ameliorates diabetes‐induced dysfunction of
the vas deferens in a rat model. Andrologia.

[r64] van Eijk IC, Peters MJ, Nurmohamed MT, van Deutekom AW, Dijkmans BA, Simsek S (2007). Decrease of fructosamine levels during treatment with adalimumab
in patients with both diabetes and rheumatoid arthritis. Eur J Endocrinol.

[r65] Venuprasad MP, Kandikattu HK, Razack S, Khanum F (2014). Phytochemical analysis of Ocimum gratissimum by LC-ESI-MS/MS and
its antioxidant and anxiolytic effects. S Afr J Bot.

[r66] Verma S, Saxena SK, Kushwaha JS, Giri R, Priyadarshi BP, Singh P (2013). Serum testosterone levels in type 2 diabetes
mellitus. JIACM.

[r67] White WE (1933). The duration of fertility and the histological changes in the
reproductive organs after ligation of the vasa efferentia in the
rat. Proc R Soc Lond B.

[r68] You Y, Ren T, Zhang S, Shirima GG, Cheng Y, Liu X (2015). Hypoglycemic effects of Zanthoxylum alkylamides by enhancing
glucose metabolism and ameliorating pancreatic dysfunction in
streptozotocin-induced diabetic rats. Food Funct.

